# Study protocol: safety and efficacy of propranolol 0.2% eye drops in newborns with a precocious stage of retinopathy of prematurity (DROP-ROP-0.2%): a multicenter, open-label, single arm, phase II trial

**DOI:** 10.1186/s12887-017-0923-8

**Published:** 2017-07-14

**Authors:** Luca Filippi, Giacomo Cavallaro, Elettra Berti, Letizia Padrini, Gabriella Araimo, Giulia Regiroli, Valentina Bozzetti, Chiara De Angelis, Paolo Tagliabue, Barbara Tomasini, Giuseppe Buonocore, Massimo Agosti, Angela Bossi, Gaetano Chirico, Salvatore Aversa, Roberta Pasqualetti, Pina Fortunato, Silvia Osnaghi, Barbara Cavallotti, Maurizio Vanni, Giulia Borsari, Simone Donati, Giuseppe Nascimbeni, Giancarlo la Marca, Giulia Forni, Silvano Milani, Ivan Cortinovis, Paola Bagnoli, Massimo Dal Monte, Anna Maria Calvani, Alessandra Pugi, Eduardo Villamor, Gianpaolo Donzelli, Fabio Mosca

**Affiliations:** 1 Neonatal Intensive Care Unit - Medical Surgical Fetal-Neonatal Department, Meyer University Children’s’ Hospital, viale Pieraccini 24, 50134 Florence, Italy; 2 Neonatal Intensive Care Unit, Fondazione IRCCS Cà Granda Ospedale Maggiore Policlinico, Università degli Studi di Milano, Milan, Italy; 3 Neonatal Intensive Care Unit, MBBM Foundation, San Gerardo Hospital, Monza, Italy; 4 Department of Pediatrics, Obstetrics and Reproductive Medicine, Neonatal Intensive Care Unit, University Hospital of Siena, Policlinico Santa Maria alle Scotte, Siena, Italy; 5 Department of Molecular and Developmental Medicine, University of Siena, Via Banchi di Sotto, 55, 53100 Siena, Italy; 6 Neonatal Intensive Care Unit, Del Ponte Hospital, Varese, Italy; 7 Neonatal Intensive Care Unit, Children’s Hospital, University Hospital “Spedali Civili” of Brescia, Brescia, Italy; 8 Pediatric Ophthalmology, A. Meyer” University Children’s Hospital, Florence, Italy; 9 Department of Ophthalmology, Fondazione IRCCS Cà Granda, Ospedale Maggiore Policlinico, Università degli Studi di Milano, Milan, Italy; 10 Department of Ophthalomolgy, ASST Monza, San Gerardo Hospital, Monza, Italy; 11 Pediatric Ophthalmology, University Hospital of Siena, Policlinico Santa Maria alle Scotte, Siena, Italy; 12 Department of Surgical and Morphological Sciences, Section of Ophthalmology, University of Insubria, Varese, Italy; 13 Department of Ophthalmology, University Hospital “Spedali Civili” of Brescia, Brescia, Italy; 14 Department of Neurosciences, Psychology, Pharmacology and Child Health, University of Florence, Newborn Screening, Biochemistry and Pharmacology Laboratory, Meyer Children’s University Hospital, Florence, Italy; 15 Laboratory “G.A. Maccacro”, Department of Clinical Sciences and Community Health, University of Milan, Milan, Italy; 16 Department of Biology, Unit of General Physiology, University of Pisa, Pisa, Italy; 17 Department of Pharmacy, “A. Meyer” University Children’s Hospital, Florence, Italy; 18 Clinical Trial Office, “A. Meyer” University Children’s Hospital, viale Pieraccini 24, 50134 Florence, Italy; 19 Department of Pediatrics, Maastricht University Medical Center (MUMC+), School for Oncology and Developmental Biology (GROW), Maastricht, The Netherlands

**Keywords:** Propranolol, Beta blocker, Proliferative retinopathy, Angiogenesis

## Abstract

**Background:**

Retinopathy of prematurity (ROP) still represents one of the leading causes of visual impairment in childhood. Systemic propranolol has proven to be effective in reducing ROP progression in preterm newborns, although safety was not sufficiently guaranteed. On the contrary, topical treatment with propranolol eye micro-drops at a concentration of 0.1% had an optimal safety profile in preterm newborns with ROP, but was not sufficiently effective in reducing the disease progression if administered at an advanced stage (during stage 2). The aim of the present protocol is to evaluate the safety and efficacy of propranolol 0.2% eye micro-drops in preterm newborns at a more precocious stage of ROP (stage 1).

**Methods:**

A multicenter, open-label, phase II, clinical trial, planned according to the Simon optimal two-stage design, will be performed to analyze the safety and efficacy of propranolol 0.2% eye micro-drops in preterm newborns with stage 1 ROP. Preterm newborns with a gestational age of 23–32 weeks, with a stage 1 ROP will receive propranolol 0.2% eye micro-drops treatment until retinal vascularization has been completed, but for no longer than 90 days. Hemodynamic and respiratory parameters will be continuously monitored. Blood samplings checking metabolic, renal and liver functions, as well as electrocardiogram and echocardiogram, will be periodically performed to investigate treatment safety. Additionally, propranolol plasma levels will be measured at the steady state, on the 10th day of treatment. To assess the efficacy of topical treatment, the ROP progression from stage 1 ROP to stage 2 or 3 with plus will be evaluated by serial ophthalmologic examinations.

**Discussion:**

Propranolol eye micro-drops could represent an ideal strategy in counteracting ROP, because it is definitely safer than oral administration, inexpensive and an easily affordable treatment. Establishing the optimal dosage and treatment schedule is to date a crucial issue.

**Trial registration:**

ClinicalTrials.gov Identifier NCT02504944, registered on July 19, 2015, updated July 12, 2016. EudraCT Number 2014–005472-29.

**Electronic supplementary material:**

The online version of this article (doi:10.1186/s12887-017-0923-8) contains supplementary material, which is available to authorized users.

## Background

### Background and rationale

Retinopathy of prematurity (ROP) is a potentially blinding disease caused by pathologic angiogenesis that occurs in the incompletely vascularized retina of preterm newborns. Despite current therapeutic strategies, ROP still represents a leading cause of potentially avoidable visual impairment and blindness in childhood. More than 30,000 preterm infants become blind or visually impaired from ROP each year worldwide [1]. In the 1940s, the so-called “first ROP epidemic” was related to the widespread use of unrestricted oxygen supplementation; the second “ROP epidemic” occurred in high-income countries in the 1970s and it was related to the increasing survival rate at lower gestational age (GA) [2–4]. In the early 1990s, an emerging epidemic of blindness due to ROP was also recorded in middle-income countries [5]. Currently, Asia is the region presenting the highest incidence of blindness due to ROP, followed by Latin America, where some countries account for an incidence of blindness/severe visual impairment related to ROP that is 2.4 times higher than in highly industrialized countries [1, 6, 7]. Therefore, the detection of a new inexpensive and easily affordable treatment strategy may be a relevant issue of global interest. Prematurity and low birth weight are the main factors associated with ROP, although other factors (i.e. respiratory failure, fetal hemorrhage, intra-ventricular hemorrhage, blood transfusions, hyperglycemia, sepsis, necrotizing enterocolitis) have been described as contributing factors to ROP development [8, 9].

Physiologically, retinal blood vessels development begins at the optic disc during the fourth month of gestation in the hypoxic uterine environment and is completed at approximately 40 weeks of gestational age. The pathogenesis of ROP has not yet been totally clarified, but the most validated hypothesis describes two different postnatal phases [10]. During the first phase, the loss of the placenta and the exposure to extrauterine relative hyperoxia are associated with low levels of Vascular Endothelial Growth Factor (VEGF) and Insulin-like Growth Factor 1 (IGF-1), resulting in a cessation of retinal vascularization [11–14]. In fact, oxygen induces retinal vasoconstriction, prevents retinal vessel growth and therefore still represents one of the main determinant of ROP development [15]. During the second phase, the retinal maturation and the development of relative hypoxia stimulate the VEGF and IGF-1 expression, causing a shift to a proliferative phase, which is characterized by an abnormal angiogenesis [16–18].

For a long time an oxygen saturation level lower than 90% has been suggested to reduce ROP risk. However, the recent demonstration that a higher oxygen saturation (91–95%) correlates with an improved survival represents an actual dilemma because, unfortunately, it induces a higher risk of ROP development [15]. Apart from oxygen tension, which is the main factor promoting the expression of angiogenic growth factors in proliferative retinopathies, other mechanisms are involved in the vascular response to ischemia/hypoxia, including the activation of inflammatory signaling pathways, oxidative stress and the production of nitric oxide [19]. Genetic factors might also affect the risk for ROP, even though no one has been identified thus far. The disease progresses more often in white than black infants and in boys than girls [20, 21].

#### The role of the β-adrenergic system

Propranolol is a non-selective β-adrenoreceptor (β-AR) antagonist. For many years, it has been largely used in the pediatric population affected by cardiovascular diseases (i.e. arterial hypertension, obstructive hypertrophic cardiomyopathy, Fallot tetralogy and arrhythmia), hyperthyroidism (i.e. neonatal thyrotoxicosis), migraine and portal hypertension with gastroesophageal varices at risk of bleeding. Propranolol is also effective and sufficiently safe in treating infantile hemangioma (IH) in childhood [22–24] and the European Medicines Agency (EMA) has recently authorized the use of propranolol for life-threatening IH, at risk of ulceration or permanent deformation. The working mechanisms of propranolol in reducing proliferative IH are not completely known and include vasoconstriction, induction of epithelial cells apoptosis and inhibition of angiogenesis [25–27]. The growth of IH is enhanced by pro-angiogenic factors, including VEGF and basic fibroblast growth factor (bFGF) and propranolol inhibits the growth of IH by decreasing the expression of pro-angiogenic factors and Hypoxia Inducible Factor 1 (HIF-1) induced by adrenergic receptors [26–32]. Some pathogenic aspects of ROP are probably common to IH, as suggested by the evidence that ROP and IH often coexist in infants weighting <1250 g [33] and that both diseases share the same histological feature. For instance, endothelia of IH and of retinal neovasculature in ROP express GLUT1 [34, 35], a factor significantly up-regulated in hypoxic tissues and stimulated by HIF-1 [36].

Additionally, as for IH, the vascular proliferative phase induced by hypoxia, which is the “second phase” in ROP pathogenesis, is promoted by VEGF. Considering that both β1 and β2-ARs are expressed in the retina [37–40], that hypoxia increases VEGF levels presumably through overactivation of the β-adrenergic system as suggested by norepinephrine accumulation in response to hypoxia [41, 42], that β-AR blockade is effective in mouse models of retinal neovascular diseases, our assumption was that the use of β-AR blockers, such as propranolol, could be useful for the treatment of ROP in infants. Indeed, several studies using a mouse model of oxygen-induced retinopathy (OIR) [43, 44] have analyzed the role of the adrenergic system in the ROP pathogenesis and the effects of β-AR antagonists and agonists on ROP development [45–47]. These studies confirmed that retinal exposure to hypoxia leads to an increase in catecholamine release, which promotes the up-regulation of pro-angiogenic factors and retinal angiogenesis by over-activating β-ARs [46]. The β-AR blockade by systemic propranolol administration decreases VEGF and IGF-1 levels, retinal hemorrhage, retinal tufts and blood-retinal barrier breakdown, improving the retinopathy score [45]. Similar findings were observed using selective β2-AR blockade [47] and after β2-AR desensitization following agonist administration [46], confirming that β2-ARs play a central role in the pathogenesis of ROP.

However, these findings obtained in C57BL/6 mice seem to conflict with results reported in 129S6 mice, a strain predisposed to develop a more aggressive neovascularization [48] and characterized by an impressive up-regulation of β3-ARs [49]. In this strain propranolol does not seem to affect the retinal response to hypoxia [49], but our hypothesis was that probably the different genetic background of the mouse strain might contribute to the different retinal responses to hypoxia [50]. The hypothesis that the insensitivity to propranolol of 129S6 mice was due to the preponderance in this strain of β3-ARs, that are minimally blocked by propranolol [51], was confirmed by the discovery that this receptor is involved in VEGF production in hypoxic retinas, through the nitric oxide pathway [52]. The discovery of a proangiogenic action of β3-ARs suggested to investigate a possible role for this receptor also in cancer growth [53–55], a new frontier of research currently for neonatologists.

#### Efficacy and safety of oral propranolol

The studies in the OIR model provided a considerable amount of results which strongly indicate that β2-AR blockade may play a significant action against hypoxia-induced retinal neovascularization. This evidence prompted an interest in exploring the possibility that the administration of propranolol may not only be used to treat IH but also be of help in the treatment of ROP. A randomized controlled trial [56] was performed to verify the efficacy and safety of oral propranolol in preterm newborns (GA < 32 weeks) with ROP stage 2 without plus in zone II [57]. Oral propranolol significantly decreased ROP progression to both stage 3 and stage 3 with plus, and none of treated newborns progressed to stage 4. The number of newborns who underwent laser photocoagulation or bevacizumab administration was significantly lower in the treated group [56]. These data are consistent with those reported by other authors [58–60]. Despite propranolol being generally safe and well tolerated in infancy, serious adverse events have been reported in unstable preterm newborns, mainly in conjunction with other conditions, such as sepsis, anesthesia or tracheal stimulation [56]. In these patients receiving the lower dose of 1 mg/kg/day, mean propranolol plasma concentration was around 20 ng/mL. Considering that pharmacological effects of β-blockers are usually related to the plasma concentrations, it appears prudent to avoid in future clinical trials propranolol concentrations higher than 20 ng/mL, that was considered a sort of safe cut-off value [56]. Although propranolol is effective in counteracting ROP progression [56, 58–60], the incidence of adverse events indicates that systemic administration is not sufficiently safe in preterm newborns [56]. Recently, also prophylactic propranolol administered on seventh day of life showed a decreasing trend in the incidence of ROP, need for laser therapy, and treatment with anti-VEGF [61].

#### Efficacy of propranolol eye drops in animal models

Since the oral administration of propranolol did not guarantee adequate safety, further experiments investigated the efficacy and safety of topical propranolol, in the form of eye drops, in animal models. In 2013, Dal Monte and co-workers demonstrated that 2% topical propranolol administration provides the retina with a drug concentration that are adequate to decrease pro-angiogenic factors (VEGF and IGF-1), retinal angiogenesis and blood-retinal barrier breakdown in OIR mice [62]. The efficacy and safety of topical propranolol were also evaluated in a rabbit model [63]. Male New Zealand white rabbits were treated with propranolol-based ocular drops at 0.1% of concentration, applied every 6 h to both eyes for 5 days. Retinal and plasma concentrations of propranolol were measured and compared with those registered after oral treatment. Despite retinal drug concentrations being similar to those reported after oral treatment, plasma propranolol levels were significantly lower after topical administration. Additionally, Draize test (a classical acute toxicity test) and cornea’s histological analysis showed no significant differences between control and treated eyes, confirming that local tolerability of ocular propranolol drops was optimal. All these findings suggested that topical propranolol formulation might be equally effective as systemic administration but have a better safety profile.

#### Safety and efficacy of propranolol 0.1% eye micro-drops in newborns

Recently an open-label, trial was performed to evaluate the safety and efficacy of propranolol 0.1% eye micro-drops in preterm newborns with stage 2 ROP without plus [64]. The study was planned according to the Simon optimal two-stage design for phase II clinical trials and it was discontinued before starting the second stage since the number of failures was above the set threshold. Even though the objective to move to the second stage was not reached, the percentage of ROP progression (around 26%) was substantially similar to that obtained after oral propranolol administration. Nevertheless, no adverse effects were observed and propranolol plasma levels were significantly lower than those measured after oral administration (consistently below the cut-off value of 20 ng/mL). Therefore, treatment with propranolol 0.1% eye micro-drops seems to be safe and well tolerated in preterm newborns, but not sufficiently effective in reducing ROP progression.

Further research is then required to identify the optimal dose and schedule of topical propranolol therapy for ROP.

### Research hypothesis

The present open-label trial is planned to evaluate safety and efficacy of propranolol 0.2% eye micro-drops in preterm newborns with stage 1 ROP without plus.

### Study objectives

#### Primary objective

To evaluate the safety and efficacy of propranolol 0.2% eye micro-drops in preventing ROP progression from Stage 1 without plus to Stage 2 with plus or 3 with plus and therefore in reducing the rate of laser treatment and rescue treatment with bevacizumab.

#### Secondary objective

To analyze the efficacy of propranolol 0.2% eye micro-drops in preventing ROP progression from Stage 1 without plus to more severe Stage ROP.

### Trial design

The present study is a multicenter, open-label, single arm phase II trial planned as a Simon optimal two-stage design [65], under the hypothesis that the treatment decreases the incidence of ROP progression to stage 3 with plus (estimated from historical data to be at least 19%) by 50% or more.

## Methods: Participants, interventions, outcomes

### Study setting

Preterm newborns delivered at GA ranging from 23 to 32 weeks and admitted to the neonatal intensive care units (NICU) contributing to the study (1. Meyer University Children’s Hospital in Florence; 2. Institute of Pediatrics and Neonatology, Fondazione IRCCS Ospedale Maggiore Policlinico, Mangiagalli e Regina Elena, Università di Milano; 3. San Gerardo Hospital in Monza; 4. University Hospital Policlinico Santa Maria alle Scotte, Siena; 5. University Hospital in Varese; 6. Children’s Hospital Spedali Civili in Brescia) were considered for enrolment.

### Inclusion criteria

The following inclusion criteria were considered:

1. Preterm newborns (GA 23–32 weeks) with birth weight < 1500 g diagnosed with stage 1 ROP in zone II or III, without plus;
2. A signed informed consent from parents.

### Exclusion criteria

1. Newborns with heart failure, congenital cardiovascular anomalies except for persistent ductus arteriosus, patent foramen ovale and small ventricular septal defects, recurrent bradycardia (heart rate < 90 beat per minute), second or third degree atrio-ventricular block, intractable hypotension, renal failure, current cerebral hemorrhage, other diseases which contraindicate the use of β-AR blockers.
2. Newborns with ROP at a more advanced stage than stage 1.
3. Newborns with aggressive posterior ROP (AP-ROP).

### Intervention

All enrolled newborns will receive propranolol as ophthalmic solution (0.2%). Three micro-drops of 6 μL propranolol solution (= 6 μg propranolol/μ-drops) will be topically applied four times daily (every 6 h) in each eye with a calibrated pipette. After propranolol administration, the nasolacrimal duct will be carefully compressed for 1 min in order to decrease the percentage of drug absorbed by the conjunctival and nasal vessels. The treatment will be started as soon as the diagnosis of stage 1 ROP without plus is confirmed and will be continued until the complete development of retinal vascularization, but for no longer than 90 days. However, ophthalmologic exams will also be performed after this period to exclude possible rebound phenomenon. In these cases, propranolol eye micro-drops treatment will be resumed until retinal vascularization is completed.

The ophthalmologic approach for newborns enrolled in the study will be in accordance with the guidelines adopted by the ETROP Cooperative Group and the AAP/AAO/AAPOS guidelines [3, 66, 67]. The RetCam Imaging System will be systematically used by ophthalmologists to evaluate ROP evolution.

Eye drops will be prepared sterilely by diluting propranolol hydrochloride powder (ACEF, Fiorenzuola d’Arda, Piacenza, Italy), in sterile water for injection at a concentration of 2%. Then, the propranolol 0.2% solution will be obtained in a horizontal laminar flow hood adding 9 ml of saline solution to 1 ml of propranolol 2% preparation.

Newborns with ROP who progressed to stage 2 plus or stage 3 plus will be treated with laser photocoagulation or intravitreal anti-VEGF (bevacizumab) administration. The ophthalmologists will choose the treatment they will consider most appropriate.

### Modification

#### Stop criteria and dose changes

Considering that unstable newborns (i.e. after anesthesia induction) have shown a high risk of adverse events (hypotension and bradycardia) due to propranolol administration, whenever surgery and/or anesthesia are indicated, the discontinuation of the propranolol eye micro-drops treatment is recommended at least 24 h before.

Newborns in whom propranolol administration will be temporarily suspended for more than two doses, with the exception of a temporary suspension before surgery will be excluded from the study.

In the case of a severe adverse event (bradycardia, bronchospasm, severe hypotension or severe local signs) due to propranolol eye micro-drops therapy, the treatment will be promptly stopped and the newborn will be excluded from the study. The concentration of propranolol will be measured on dried blood spots to verify the relationship between the adverse event that occurred and the plasmatic levels of propranolol. Moreover, after the first adverse event, the study could be restarted reducing the propranolol eye drops dosage to two micro-drops of 6 μL 0.2% propranolol solution administered four times daily in each eye. An additional enrolment phase will be opened and will be based on a new study population not including newborns previously treated.

Similarly, the study could be restarted increasing the concentration of propranolol eye drops solution up to 0.3% in case of treatment failure in terms of efficacy during the first stage of the study, if plasma propranolol concentrations are below the cut off of 20 ng/ml.

The outcomes of infants who develop adverse effects to propranolol will be reported to Pharmacovigilance Center and then published.

## Methods: Data collection, management, analysis

### Data collection methods

All data will be registered in specific case report form including neonatal demographic data, prenatal and perinatal history and morbidity profiles of both mother and newborn. Hemodynamic parameters, diuresis and respiratory parameters will be continuously monitored during the first 3 weeks of treatment. Biochemical parameters, such as a complete blood count, serum electrolytes levels, renal and liver function tests will be measured before starting treatment (T0) and once a week for the first 3 weeks of treatment (T7, T14, and T21). Electrocardiogram and echocardiogram will be performed before starting treatment and once a week for 3 weeks of treatment. Any drugs that are concomitantly administered and procedures performed will be recorded.

To investigate the safety of propranolol 0.2% eye micro-drops treatment, the concentration of propranolol will be measured on dried blood spots using the liquid-chromatography tandem-mass spectrometry test [68, 69] at the steady state on the 10th day of treatment, before administering therapy (T0), after 2 (T2), 4 (T4) and 6 h (T6). Additionally, parents will be asked to consent to us taking and storing 0.3 ml of plasma.

The stage of ROP disease should be established by complete ophthalmological evaluations, planned according to ROP Guidelines [3, 66, 67], also considering the progression and the severity of ROP. The ophthalmologic exam should verify the absence of local adverse events due to the propranolol eye micro-drops treatment, as well as analyze corneal and vitreous transparencies, lens opacity, and regression of vessels in the tunica vasculosa lentis. The ROP progression will be monitored by indirect ophthalmoscopy using a 20D and 28D lens. The RetCam Imaging System will be systematically used by ophthalmologists to evaluate ROP evolution.

The timeline of the study is reported in Additional file 1.

All the adverse effects will be notified to the qualified responsible of pharmacovigilance, using the specified report form.

### Statistical methods

#### Preliminary analysis

To plan the present multicenter, open-label, single arm, phase II trial, a preliminary analysis was performed to evaluate historical ROP incidence in the 6 NICUs involved in the study.

This analysis included all preterm newborns admitted to the NICUs contributing to the study (Florence, Milan, Monza, Siena, Varese, Brescia) and diagnosed with any stages of ROP from 2011 to 2015. During the 5 years preceding the present study, 248 patients out of 2165 very low birth weight newborns (11.5%) were diagnosed with ROP. Demographic and obstetric characteristics of this historical cohort are reported in Table 1. However, only 237 of these newborns (95.6%) shared the same enrollment criteria of this planned trial. In fact, 3 newborns were suffering from AP-ROP and 8 newborns showed a ROP ≥ stage 2 at first examination. Therefore, in Table 1 we also show the demographic and obstetric characteristics of these 237 newborns who showed ROP stage 1 at first examination.

**Table 1:** Demographic and obstetric characteristics of historical cohort, co-morbidities and co-interventions

Demographic and obstetric characteristics	Any stage ROP	Stage 1 ROP at first visit
Newborns*, n* (%)	248	237 (95.6)
Gestational age, weeks, *mean ± SD*	26.6 ± 2.0	26.7 ± 2.0
Birth weight, g, *mean ± SD*	838 ± 233	843 ± 235
Male, *n* (%)	129 (52.0)	126 (53.2)
Caesarean delivery, *n* (%)	170 (68.5)	162 (68.3)
Stained amniotic fluid, *n* (%)	18 (7.3)	16 (6.7)
Apgar Score, 1 min, *mean ± SD*	4.6 ± 2.3	4.6 ± 2.3
Apgar Score, 5 min, *mean ± SD*	7.4 ± 1.7	7.4 ± 1.7
Post menstrual age at diagnosis, weeks, *mean ± SD*	34.1 ± 2.2	34.2 ± 2.3
Co-morbidities and co-intervention		
Respiratory distress syndrome, *n (%)*	239 (96.4)	228 (96.2)
Surfactant treatment, n (%)	208 (83.9)	198 (83.5)
Duration of oxygen exposure (days), *median (range)*	49.4 (0–291)	46.7 (0–291)
Bronchopulmonary dysplasia ^a^, *n* (%)	170 (68.5)	160 (67.5)
Candida sepsis, *n* (%)	12 (4.8)	12 (5.1)
Other sepsis, *n* (%)	143 (57.7)	132 (55.7)
Number of red blood cell transfusions, *median (range)*	5 (0–19)	5 (0–19)
Intraventricular hemorrhage, grade 3–4, *n* (%)	40 (16.1)	38 (16.0)
Post-hemorrhagic hydrocephalus, *n* (%)	17 (6.8)	15 (6.3)
Cholestasis, *n* (%)	66 (26.6)	60 (25.3)
Necrotizing enterocolitis, *n* (%)	32 (12.9)	31 (13.1)
Gastrointestinal perforation, *n* (%)	26 (10.5)	25 (10.5)
Surgical closure of patent ductus arteriosus, *n* (%)	56 (22.6)	52 (21.9)
Survival, n (%)	245 (98.8)	234 (98.7)

A total of 63 newborns out of 248 diagnosed with any stage of ROP (25.4%) showed a stage 2 or 3 with plus and received a treatment (Table 2). The same analysis was repeated excluding the three newborns suffering from AP-ROP, and the eight newborns who showed a ROP stage ≥2 at first examination. Regarding the 237 newborns that showed ROP stage 1 at first examination, 58 (24.5%) progressed from stage 1 to stage 2 or 3 with plus. Overall, 45 newborns underwent laser photocoagulation, while 27 newborns were treated with bevacizumab administration (14 newborns, in fact, received both treatments). Four patients progressed to stage 4 ROP and were treated with vitrectomy (one also with cryotherapy). Finally, one newborn progressed to stage 5 ROP. These data were used to plan the current prospective study.

**Table 2:** Ophthalmologic outcome of historical cohort

ROP progression	Any stage ROP	Stage 1 ROP at first visit
Newborns*, n* (%)	248	237 (95.6)
Aggressive Posterior ROP, *n* (%)	3 (1.2)	
Stage ≥2 at first examination, *n* (%)	8 (3.2)	
Stage 1 ROP at first examination, *n* (%)	237 (95.6)	
Stage 2, *n* (%)	172 (69.3)	165 (69.6)
Stage 3, *n* (%)	72 (29.0)	68 (28.7)
Stage 2 or 3 ROP with plus, *n* (%)	63 (25.4)	58 (24.5)
Stage 4 ROP, *n* (%)	4 (1.6)	4 (1.7)
Stage 5 ROP, *n* (%)	1 (0.4)	1 (0.4)
Treatment with laser photocoagulation, *n* (%)	47 (18.9)	45 (19.0)
Treatment with bevacizumab, *n* (%)	30 (12.1)	27 (11.4)
Vitrectomy, *n* (%)	4 (1.6)	4 (1.7)
Cryotherapy, *n* (%)	1 (0.4)	1 (0.4)

### Endpoint

For the present multicenter, open-label, single arm, phase II trial, the following endpoint will be evaluated:

### Primary endpoint

-Number of infants who progress from ROP Stage 1 in zone II or III, without plus to Stage 2 with plus or Stage 3 with plus.
-Analysis of propranolol plasma concentration at the steady state (on the tenth day of treatment).

### Secondary endpoint

-Number of infants who progress to Stage 2 without plus ROP.
-Number of infants who progress to Stage 3 without plus ROP.
-Number of infants who progress to Stage 4 or 5 ROP with total or partial retinal detachment.
-Number of infants who need vitrectomy.
-Number of adverse events due to propranolol eye drops treatment.

### Experimental plan

**Fig. 1:**
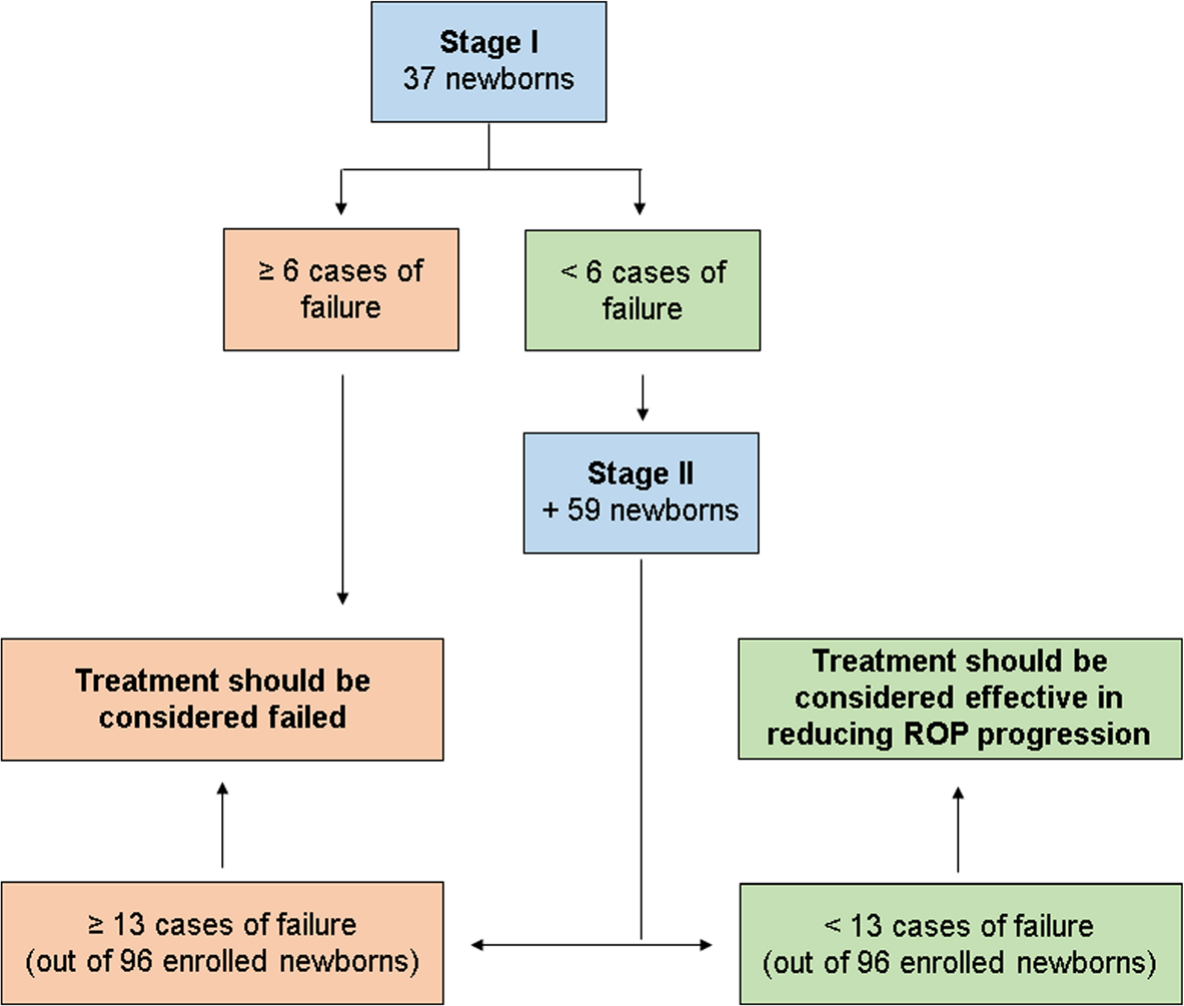
Simon optimal two-stage design for phase II clinical trials

The study was planned as a Simon optimal two-stage design [65] (Fig. 1), under the hypothesis that propranolol 0.2% eye micro-drops treatment decreases the incidence of ROP progression to stage 2 or 3 with plus by at least 50%. From a first analysis of the historical cohort of neonates the incidence was found to be 19%. A second and deeper analysis showed that the incidence was likely somewhat higher (24.5%) (Table 2). The study size adopted is based on the first and more conservative estimate. Therefore, considering an alpha error of 0.05 and a power of 80%, the treatment should be considered failed if:

-at least 6 cases of failure (a progression of ROP to stage 2 with plus or 3 with plus) is observed in the first 37 newborns enrolled;
-at least 13 out of 96 newborns enrolled show a treatment failure (a progression of ROP to stage 2 with plus or 3 with plus).

At the end of the study, if the overall cases of failure are less than 13 out of 96 newborns, the treatment with propranolol 0.2% eye drops will be considered effective in decreasing the rate of ROP progression to stage 2 or 3 with plus.

Additionally, considering the serious adverse effects observed in newborns receiving oral propranolol with plasma concentrations around 20 ng/mL, this value is currently considered a sort of safe cut-off value [55]. For this reason, if the mean propranolol plasma concentration will be less than 20 ng/ml, as expected, the treatment with propranolol eye drops should be considered safe, being unable to cause high plasma levels of propranolol.

The 96 preterm newborns will be enrolled approximately in 2–3 years. The enrokllment will be competitive: the individual centers participating in the trial will not have a predetermined number of patients to recruit, but they will compete with each other to recruit all expected patients. The trial will be completed when the last newborn enrolled has completed the treatment schedule or achieved final retinal vascularization.

## Ethics

### Research ethics approval

The phase II study entitled “Study protocol: Safety and efficacy of propranolol 0.2% eye drops in newborns with retinopathy of prematurity: a phase II study (DROP-ROP-0.2%)” has been ethically approved by the Ethics Committees of centers involved in the trial and by the Italian Medicines Agency (AIFA/RSC/P/59172). Approval was obstained from Comitato Etico Pediatrico Regione Toscana (for Meyer University Children’s Hospital of Florence, and for University Hospital Policlinico Santa Maria alle Scotte, Siena), from Comitato Etico Milano Area B (Institute of Pediatrics and Neonatology, Fondazione IRCCS Ospedale Maggiore Policlinico, Mangiagalli e Regina Elena, Università di Milano), from Comitato Etico della Provincia Monza Brianza (San Gerardo Hospital in Monza), from Comitato Etico Provinciale di Varese (University Hospital in Varese) and from Comitato Etico della Provincia di Brescia (Children’s Hospital Spedali Civili in Brescia). Whenever a newborn meets the inclusion criteria, parents should be informed on the aim, the procedures and the risks of the study. Then, signed parental informed consent is to be obtained from a physician responsible of the study prior to the enrolment.

## Discussion

The aim of the present study is to evaluate the therapeutic role of propranolol 0.2% eye micro-drops in newborns with a precocious stage of ROP. Treatment with oral propranolol is effective in preventing ROP progression in preterm newborns, but appears unsafe. Furthermore, data from a previous trial suggested that propranolol 0.1% eye micro-drops had an optimal safety and tolerability profile in preterm newborns, although efficacy in reducing ROP progression was not sufficient. The optimal dosage and concentration of propranolol eye drops to use in preterm newborns are still uncertain. However, considering the optimal safety profile of propranolol 0.1% eye micro-drops, it is likely that this dosage could be increased without compromising safety. Similarly, the excellent local tolerability also suggests that the concentration of propranolol solution could be increased without the risk of local adverse reactions. According to these considerations, the present protocol study plans to increase both dosage and concentration of propranolol eye drops in order to improve the efficacy profile. Additionally, the optimal time to start propranolol treatment has not yet been clarified. In the previous study with 0.1% eye micro-drops, the treatment was started at an advanced stage of ROP (stage 2 without plus), a stage that is quite close to the threshold of ophthalmological treatment. Therefore, we assumed that starting therapy at an earlier stage of ROP (stage 1) could represent an additional advantage.

Finding the optimal dosage and schedule of propranolol eye micro-drops treatment could represent a crucial turning point in ROP therapy. In fact, propranolol eye drops is apparently a safe, inexpensive and easily affordable treatment. Considering also the high prevalence of ROP registered in middle income countries, these advantages become even more relevant.

## Additional file

Additional file 1: BMC Pediatrics Appendix 1. Study timeline. Appendix 1 reports the timeline of the study. (DOC 50 kb)

## Abbreviations

AP-ROP: Aggressive posterior ROP
bFGF: Basic fibroblast growth factor
EMA: European Medicines Agency
GA: Gestational Age
HIF-1: Hypoxia Inducible Factor 1
IGF-1: Insulin-like Growth Factor 1
IH: Infantile hemangioma
NICU: Neonatal intensive care units
OIR: Oxygen-induced retinopathy
ROP: Retinopathy of prematurity
VEGF: Vascular Endothelial Growth Factor
β-AR: β-adrenoreceptor
