# Supplementary material for: Study protocol: safety and efficacy of propranolol 0.2% eye drops in newborns with a precocious stage of retinopathy of prematurity (DROP-ROP-0.2%): a multicenter, open-label, single arm, phase II trial
Source: BMC Pediatr. 2017 Jul 14;17:165. doi: 10.1186/s12887-017-0923-8 (PMC5513165; doi:10.1186/s12887-017-0923-8)
Supplement: Additional file 1: — BMC Pediatrics Appendix 1. Study timeline. Appendix 1 reports the timeline of the study. (DOC 50 kb) [file 12887_2017_923_MOESM1_ESM.doc]

**Appendix 1. Study timeline**

|  | **Screening** | **Enrolment** |  | | | | |
| --- | --- | --- | --- | --- | --- | --- | --- |
| **TIMEPOINT** |  | **T_0_** | **T_7_** | **T_10_** | **T_14_** | **T_21_** | **T_90_*** |
| **Ophthalmologic screening:** | **X** |  |  |  |  |  |  |
| **ENROLMENT:** |  |  |  |  |  |  |  |
| **Eligibility screen** |  | **X** |  |  |  |  |  |
| **Informed consent** |  | **X** |  |  |  |  |  |
| **Allocation** |  | **X** |  |  |  |  |  |
| **INTERVENTIONS:** |  |  |  |  |  |  |  |
| **Propranolol eye micro-drops 0.2%** |  |  |  |  |  |  |  |
| **ASSESSMENTS:** |  |  |  |  |  |  |  |
| **Plasma propranolol levels** |  |  |  | **X** |  |  |  |
| **Ophthalmologic visit** |  |  |  |  |  |  |  |
| **Biochemical samplings** |  | **X** | **X** |  | **X** | **X** |  |
| **Plasma storage** |  | **X** | **X** |  | **X** | **X** |  |
| **Hemodynamic parameters** |  |  |  |  |  |  |  |
| **Respiratory parameters** |  |  |  |  |  |  |  |
| **Cardiac ultrasonography** |  | **X** | **X** |  | **X** | **X** |  |
| **Electrocardiogram** |  | **X** | **X** |  | **X** | **X** |  |

* or complete vascularization of retina
